# HCVIVdb: The hepatitis-C IRES variation database

**DOI:** 10.1186/s12866-016-0804-6

**Published:** 2016-08-15

**Authors:** Evan W. Floden, Anas Khawaja, Václav Vopálenský, Martin Pospíšek

**Affiliations:** 1 Department of Genetics & Microbiology, Faculty of Science, Charles University in Prague, Viničná 5, 128 44 Prague 2, Czech Republic; 2 Centre for Genomic Regulation (CRG), The Barcelona Institute of Science and Technology, Dr. Aiguader 88, Barcelona, Spain; 3 Universitat Pompeu Fabra (UPF), Barcelona, Spain

**Keywords:** Hepatitis C, HCV, Internal ribosome entry site, IRES, Translation efficiency, Database

## Abstract

**Background:**

Sequence variability in the hepatitis C virus (HCV) genome has led to the development and classification of six genotypes and a number of subtypes. The HCV 5′ untranslated region mainly comprises an internal ribosomal entry site (IRES) responsible for cap-independent synthesis of the viral polyprotein and is conserved among all HCV genotypes.

**Description:**

Considering the possible high impact of variations in HCV IRES on viral protein production and thus virus replication, we decided to collect the available data on known nucleotide variants in the HCV IRES and their impact on IRES function in translation initiation. The HCV IRES variation database (HCVIVdb) is a collection of naturally occurring and engineered mutation entries for the HCV IRES. Each entry contains contextual information pertaining to the entry such as the HCV genotypic background and links to the original publication. Where available, quantitative data on the IRES efficiency in translation have been collated along with details on the reporter system used to generate the data. Data are displayed both in a tabular and graphical formats and allow direct comparison of results from different experiments. Together the data provide a central resource for researchers in the IRES and hepatitis C-oriented fields.

**Conclusion:**

The collation of over 1900 mutations enables systematic analysis of the HCV IRES. The database is mainly dedicated to detailed comparative and functional analysis of all the HCV IRES domains, which can further lead to the development of site-specific drug designs and provide a guide for future experiments. HCVIVdb is available at http://www.hcvivdb.org.

**Electronic supplementary material:**

The online version of this article (doi:10.1186/s12866-016-0804-6) contains supplementary material, which is available to authorized users.

## Background

Although the hepatitis C virus (HCV) is an important pathogen infecting between 150 and 200 million people worldwide, the existence of the virus was not proven until 1989 [1, 2]. Hepatitis C virus often develops chronic infections with a long asymptomatic initial phase, which can, however, result in liver cirrhosis and cancer. The standard therapy for treatment of HCV in patients comprises a combination of pegylated interferon (peg-IFN) and the nucleoside analogue ribavirin. This is currently being complemented with several direct-action antivirals targeting viral proteases, polymerase and/or helicase. However, efficiency of either treatment is dependent on the HCV genotype, and resistant viruses have appeared almost concurrently with introduction of the new antivirals on the market [3, 4].

HCV is a single stranded positive-sense RNA virus from the genus *Hepacivirus*, a member of the *Flaviviridae* family. Phylogenetic studies have suggested six genotypes of HCV with several subtypes within each of them. It is thought that all of the genotypes share a common ancestor 300–400 years ago [5]. Whereas there are significant variations within the protein-coding segment of the genome, the 5′ UTR containing the internal ribosome entry site (IRES), which is responsible for viral genome translation, is relatively strongly conserved among all genotypes.

The HCV IRES spans a region of ~341 nucleotides and is composed of structurally distinct domains I, II, III and IV [6, 7]. Both sequence and structural conservation of HCV IRES are important to maintain its direct and functional contacts with the translational machinery and deliver an optimal yield of viral protein synthesis. Recent cryo-electron microscopy (cryo-EM) and molecular modelling experiments further advanced our knowledge on molecular interactions between the HCV IRES and ribosomes and our understanding of coordinated structural rearrangements within the HCV IRES and associated complexes, which are crucial for translation initiation [8–11] The close relationship between HCV IRES structure and function has also been reviewed recently [12].

The analysis of HCV IRES mutation data and the effects of mutations on translational efficiency is not a simple task. The data generated from thousands of experiments are spread across many journal articles, with no standardized reporting format. Information has often been presented within figures, severely limiting computational parsing and subsequent analysis. Prior to the development of the HCVIVdb, there was no central repository for the various mutations observed within the HCV IRES. We have developed a syntax for collating this information and, to date, have generated a dataset containing 1564 entries comprising 1967 sequence variations. The collected data have been characterized in multiple categories that assist the users in conducting comparative and functional analyses among various HCV IRES regions.

## Construction and content

All HCV IRES entries were manually gathered from the majority of published studies that dealt with modifications in the HCV IRES either occurring naturally in HCV patients subjected and/or not subjected to antiviral therapy or with modifications intentionally introduced to the viral genome by in vitro mutagenesis. The aim of most of these individual studies was to evaluate the impact of HCV IRES variants on the translation of the viral RNA genome and/or its replication. The entries obtained manually from these studies were arranged to reflect the availability of published information including HCV genotype, nucleotide changes, systems used to monitor translation efficiency, activity in translation assays, plasmid constructs and reporter genes used, clinical data and the original publication reference. Entries were aligned with the respective HCV reference genome of the particular study (Table 1).

**Table 1:** Assembly of HCV IRES data collected from publications contains various fields/experimental parameters to study various aspects of viral translation. Example is from Barria et al. [22]

Data classification	Example
Mutation(s)	U 80 C
Translational activity	52 ± 5
HCV genotype	1b
Reporter genes	RLuc/FLuc
Translational system	Rabbit reticulocyte lysate (RRL)
Natural or Engineered	Natural
Notes	19 patients, naïve to antiviral treatment
Citation	Barria et al. [22]
Other	Reference sequence: Genotype 1b (AJ238799.1)

**Fig. 1:**
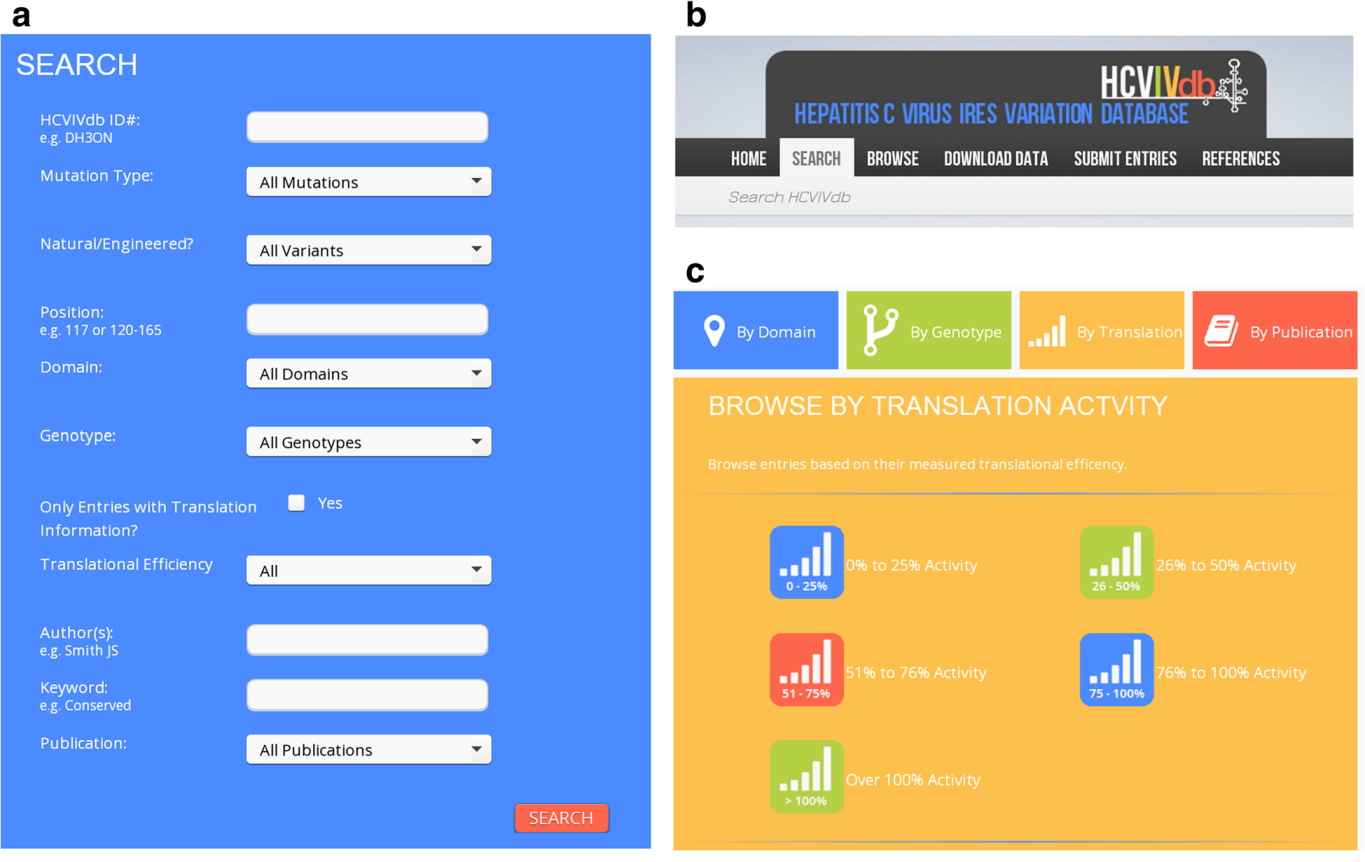
An overview of the HCVIVdb **a**) A display of the basic search that permits users to find and sort data using multiple criteria **b**) A title banner: searching, browsing, downloading and submitting data are all readily available. **c**) Data can be browsed according categories such as domain, subdomain, genotype and translational efficiency to which they pertain

The architecture of the HCVIVdb was designed to be efficient and user-friendly to maximize utilization of the data and their application by users. For this purpose, the manually collected variations of the HCV IRES were further classified into different categories for easier access to information and wide-range analysis. The search engine provides users with access to all HCV IRES mutation types ranging from point and multiple substitutions to insertions and deletions. Each entry has been given a unique ID and all entries have been divided into the two main distinct groups of naturally occurring and engineered variations (Fig. 1). A nomenclature was adopted whereby e.g. U 80 C denotes a single nucleotide substitution where uracil is replaced by cytosine at position 80. To describe the HCV IRES structure, a designation ‘domain’ was used to represent all the domains and subdomains with proper numbering (domain II, III, IIIa, IIIb etc.). Entries were further classified according to the respective variations’ presence in HCV IRES domain I through IV, their genotype, the measured activity in a translation assay, etc. All entries are available with respective publication references and direct links to the PubMed ID. This permits users to access the original source where the entry was reported and retrieve any further required details.

**Fig. 2:**
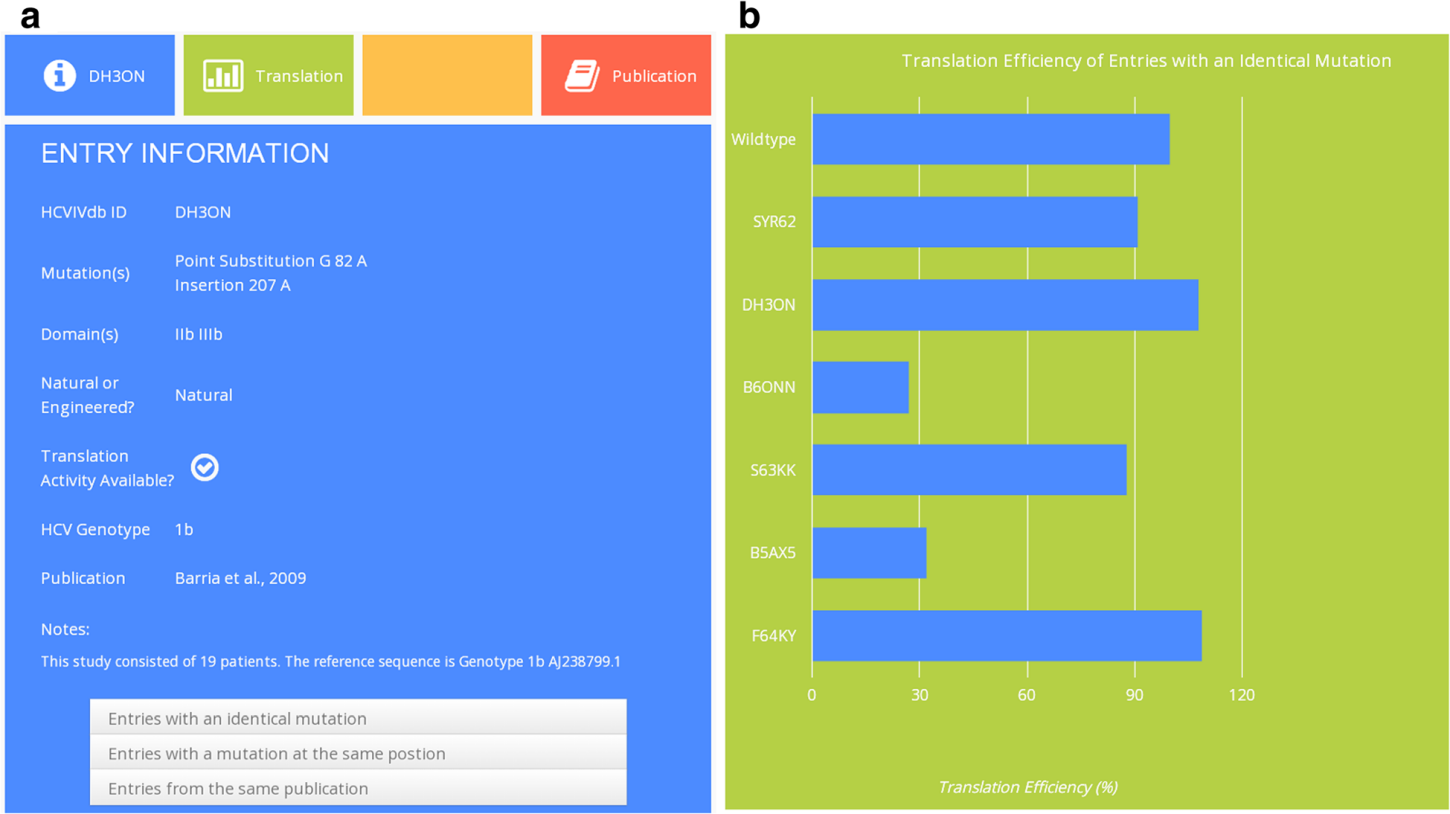
The results of the example query. The query was a double mutation in domain IIb and IIIb of the HCV IRES. **a**) The panel shows all information available for the HCVIVdb ID depicted. The website immediately offers also an interactive list of all entries containing either the same mutation(s) or mutation(s) in the same position of the HCV IRES. **b**) HCVIVdb generates a graphical display of the translational activities of the entries containing a mutation(s) identical with the entry retrieved by the search engine

The analytical tools available on the database website allow for browsing through variation entries grouped according to individual domains and sub-domains (10 categories) of the HCV IRES, according to their genotypes and according to the original publication. Entries containing data regarding translational activity can be browsed within five categories differing in a range of the measured translational efficiency relative to the wild type control in the respective experiments. Users can easily and quickly reach all available HCV IRES variations falling into one of the following categories of translational efficiency: 0–25 %, 26–50 %, 51–76 %, 76–100 % and over 100 % of the wild type. This option allows for quick elucidation of regions more or less sensitive to individual and/or multiple IRES modifications. The database contains an extensive search tool as well, which allows users to search through the entries according to one or more of the following parameters simultaneously: HCVIVdb ID, mutation type (point substitution, any substitution, insertion and deletion), whether the variation is natural or engineered, nucleotide position and/or nucleotide range within the IRES, domains and subdomains, range of translational efficiency, author, keywords and original publication (Fig. 1). The entries have been organized so that searching for a distinct variation allows evaluation of other entries with identical mutations along with mutations at the same location. The selection of these supportive mutations can further highlight the information regarding their function, genotype and other experimental parameters (Fig. 2).

The data are stored in a MySQL database made up of several related tables enabling fast and efficient data access via the web interface. A standard syntax ensures efficient and accurate parsing of data and allows searching based on relevant criteria. The user interface was designed to have a simple and easy-to-navigate structure with key elements including search and results pages. The search engine allows for targeted queries relevant to the user. The results are displayed in a real time as dynamically generated tables and graphs using the Google Charts Application Programming Interface (API). HCVIVdb is available at the web address http://www.hcvivdb.org.

## Utility and discussion

The underlying objective for the development of HCVIVdb was to gain an insight into the behavior and mechanics of the HCV IRES. The natural and engineered variations in the domains (I-IV) of the HCV IRES can impact the efficiency of its translation; therefore, the compilation of both kinds of mutations provided by the database can facilitate targeted drug design. The main aim, however, is to conduct a detailed comparative analysis of the variability in different regions of the HCV IRES in relation to its function. With the availability and characterization of the data into various categories, HCVIVdb allows users to analyze the impact of nucleotide changes on HCV IRES-mediated translation by their respective domains, by genotypes and by the range of translation efficiency (Fig. 1).

**Fig. 3:**
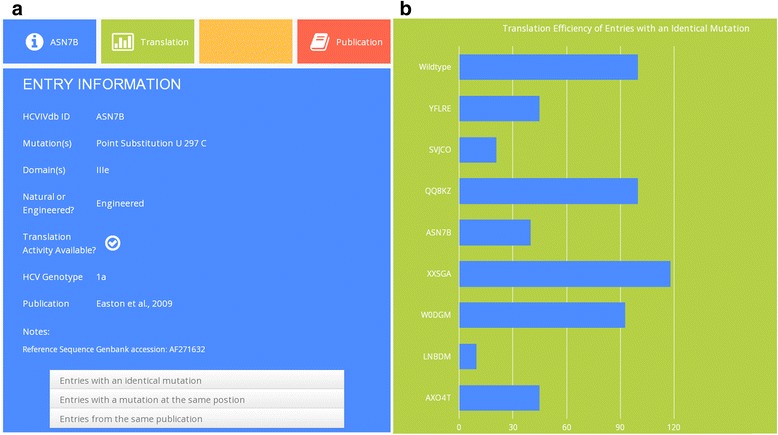
The translational efficiency of HCV IRESs containing mutations at nucleotide U297. Significant variations in activities of the individual HCV IRESs were found while comparing samples from different studies. The graph also represents double mutations and their respective HCV IRES translation efficiencies

In the context of the comparative analysis of available data, we also discovered some of the mutants reported in different studies, with nucleotide variations in the same position but varying translational efficiencies. Analysis of variations at nucleotide position 297 can serve as such an example underlining usefulness of the HCVIVdb for comparative analyses of the impact of HCV IRES variations on its function. The translational activity of mutants with a point substitution at location 297 was found to vary, displaying a response that ranges from a significant decrease in activity to a complete restoration in efficiency, relative to the wild type (Fig. 3).

The proposition of nucleotide U297 forming a Watson-Crick base pair with a bulged-out A288 [13] was consistent with the crystal structure, which showed a looped-out U297 base pairing with A288 revealing a double-pseudoknot [14]. Several studies investigating HCV IRES performance upon substitution of nucleotide U297 showed decreased activity of the mutated IRES as assayed in rabbit reticulocyte lysate (RRL), HeLa and/or Cos-7 cells [13–16]. However, similar point mutations introduced at nucleotide U297 by the Doudna laboratory exhibited responses of the HCV IRES that are mostly similar to the wild type [14]. The introduction of compensatory mutations at A288-U297 interhelical base pair (HCVIVdb ID: QQ8KZ [13], W0DGM [14], both containing two simultaneous substitutions A 288 G and U 297 C, Fig. 3b) restored translational efficiency to nearly that of the wild type [13, 14], whereas mutations with altered purine/pyrimidine pairing of the interhelical base pair and/or purine at nucleotide 297 showed reduction in activity [14]. Interestingly, decreased activity of the HCV IRES carrying U 297 C and/or U 297 A substitution was more profound when assayed in living cells [15, 16] than in RRL [13, 14]. In comparison to the double mutant (HCVIVdb ID: QQ8KZ) at A288-U297 interhelical base pair, which showed toe-print stops similar to the wild type at positions G318 and G319 (stem I of the pseudoknot) in both 48S and 80S, the single mutants A 288 G and U 297 C did not display stops at these locations. This suggests that single mutants interfere with tertiary interactions near the pseudoknot, which may disrupt the functional outcome of translation [13].

### Validity of HCVIVdb

**Fig. 4:**
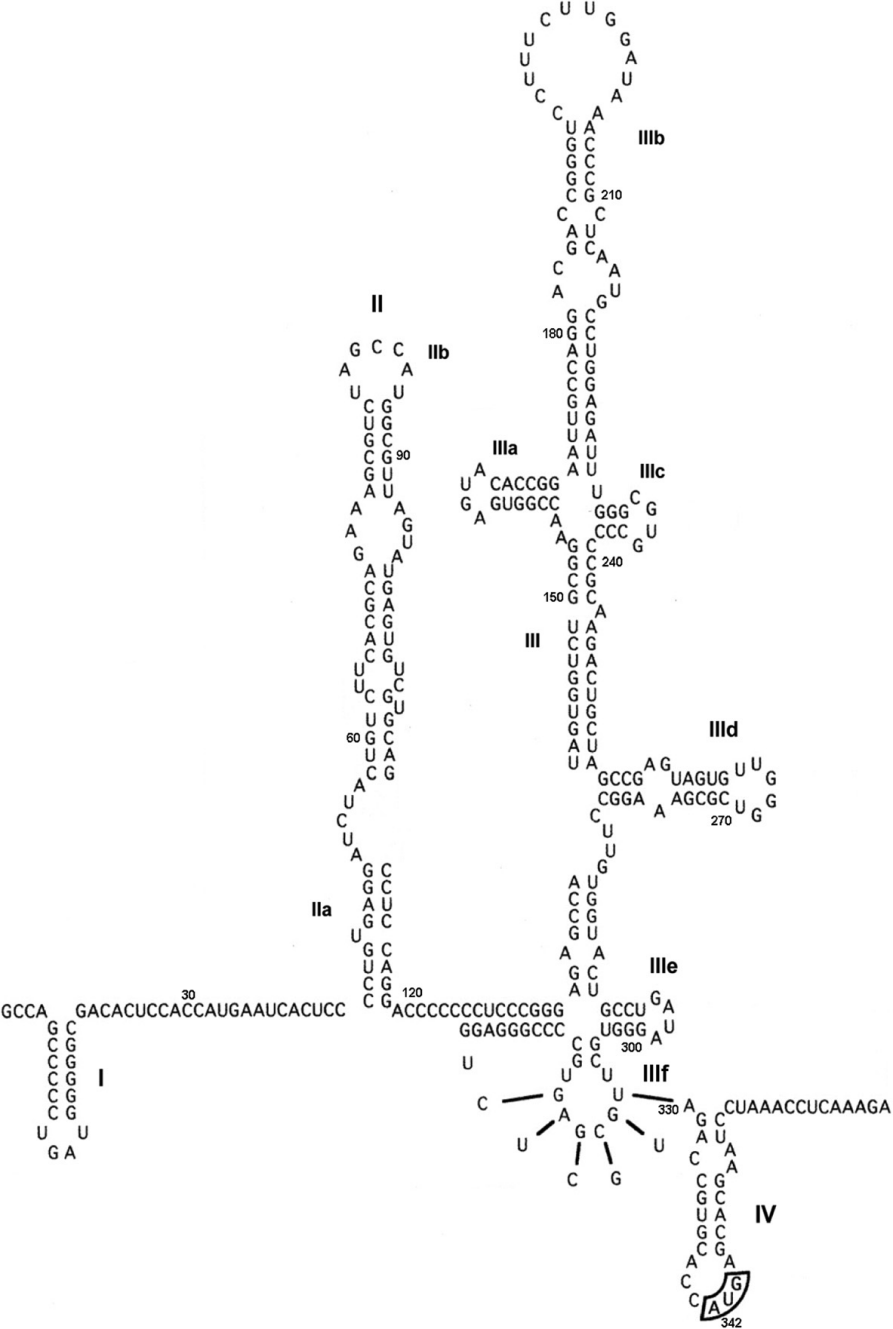
The proposed HCV IRES secondary structure (domain II-IV) [31, 32]

To validate the individually reported mutations in HCVIVdb, we compared the data with our analysis of the variation frequency at each nucleotide position in a set of over 2000 full-length HCV genome sequences from the NIAID Virus Pathogen Database and Analysis Resource (ViPR) [17]. All sequences were aligned using the MUSCLE [18] multiple sequence alignment (MSA) software and the frequency of occurrence of each nucleotide at each position of the HCV IRES (1–356 nucleotides) was counted. The results were compared with the variability of HCV IRES as obtained from the natural entries of the HCVIVdb. Both obtained datasets are in good agreement. Hyper-variable positions in the multiple sequence alignment of the full-length ViPR sequences corresponded to the highest number of the naturally occurring mutations in HCVIVdb, whereas conserved positions corresponded to the fewest number of mutations in HCVIVdb. Among the top three hypervariable nucleotide counts in the HCVIVdb and ViPR were nucleotides 204, 243 and 183. Nucleotide 204 is located in the upper loop of domain IIIb, whereas nucleotide 243 occurs in the stem III region of the HCV IRES (Fig. 4).

The nucleotide found at location 204 is mainly cytosine (C) or adenine (A). The presence of a uracil (U) base was insignificant, and there was almost no guanine (G) located at position 204 in both the HCVIVdb and the multiple sequence alignment. This nucleotide has been shown to be protected from RNase ONE™ ribonuclease cleavage upon attachment to the eukaryotic initiation factor 3 (eIF3) [19]. Further, HCV IRES translation efficiency does not seem to be affected by the observed nucleotide changes at this position (Table 2) [20–23]. The majority presence of adenine and cytosine in various HCV genotypes at this location suggests the evolutionary preservation of nucleotide 204 with A, C, and U having little impact on translational activity. The absence of guanine may suggest structural stability that may not be favorable for IIIb loop conformation and results in a loss of translational response. Another possibility might be that guanosine at position 204 might interfere with eIF3 and/or any *trans*-acting factor biding. However, all these hypotheses are pure speculations because no experimental data are available for G at position 204.

**Table 2:** An example of translational efficiencies of HCV IRESs containing variations of some hypervariable and/or conserved nucleotides as revealed from the HCVIVdb data analysis

Hypervariable nucleotides	Translational activity %	References
A 204 C	~100	[20]
C 204 U	109 ± 9	[22]
C 204 U	~80	[21]
U 204 A	Retains full activity	[23]
A 243 G	92	[20]
G 203 A + G 243 A	100	[20]
C 204 A + G 243 A	83	[21]
Conserved nucleotides	Translational activity %	References
G 266 A	5	[25]
G 266 C	3	[23]
G 267 C	>95 reduction in activity	[24]
G 267 C	2	[23]
G 268 C	>95 reduction in activity	[24]
G 268 C	3	[23]

We also came across nucleotides in various HCV IRES regions that were entirely conserved and where mutational changes in these nucleotides induced a devastating translational response. Most of the regions that display more than 90 % sequence conservation either interact directly with the translational machinery or are needed for maintenance of the IRES structural configuration, which is critical for HCV IRES activity. One such region is in domain IIId of the HCV IRES (266–268) consisting of the G triplet (Fig. 4). Functional and structural studies have shown an interaction of the (266–268) GGG in domain IIId with the 40S subunit, and any nucleotide changes decrease viral translational efficiency drastically (Table 2) [23–25]. The (266–268) GGG sequence in domain IIId has been shown to contact 18S rRNA through a (1116–1118) CCC sequence in the apical loop of expansion segment 7 (ES7) with complementary base pairing, as analyzed through dimethyl sulphate (DMS) modification [26] and also demonstrated functionally [27]. A cryo-EM structure of the HCV-IRES bound to 40S ribosome at 3.9Å has also displayed specific contact sites of the HCV IRES domain IIId loop forming a kissing complex within the apical loop of ES7, reinforced by the interaction with domain IIIe [8, 10]. Recently, structure probing techniques such as selective 2′-OH acylation analysed by primer extension (SHAPE) and footprint analysis together with molecular modelling were employed to visualize and reveal the contact sites of domain IIId loop and the 18S rRNA. Interaction of wild type HCV IRES and the IIId loop mutants with 40S investigated through structural probing alongside 3D model led to the conclusion that domain IIId loop interacts directly with the ribosomal helix 26 of ES7 and is crucial in coordinated structural re-arrangements of HCV IRES/18S rRNA upon formation of a binary complex that facilitates HCV mRNA translation [9]. The extreme conservation of these nucleotide sequences have also been observed in the MSA which exhibits almost 100 % preservation of GGG nucleotides in all 2006 HCV genome entries.

### Comparison with other databases

The importance of the hepatitis C virus as a threat to human health and its enormous variability has led to the creation of specialized public databases serving both as a data repositories and tools to compare, align and analyse viral sequences and other HCV-related data. These databases are designed to focus on different areas of the HCV, including the sequence variability, phylogeny, protein structure and immunology. However, many of these databases are rather old and have not been updated for years.

One such database is the European hepatitis C virus database (euHCVdb) designed to analyze the genetic variability of the HCV genome through a collection of computer-annotated HCV sequences based on reference genomes. The well-characterized reference genome of 26 HCV sequences representing 18 subtypes provides fully automated standardization of nomenclature for all entries with further description of the genome and proteins along with the genotype, references, cross-references to other databases, genomic regions and the source of the sequence. However, some of the tools are no longer functional, and the last database update is from January 2011 [28].

Another such database, the Los Alamos Sequence and Immunology Database, was modeled upon an HIV database that permits for storage of large sequence sets in the database along with dynamic alignment [29]. The database has been designed for users to align and evaluate HCV sequence data that are deposited in GenBank. The information may include genotype, subtype, sampling country and year, isolate names, etc. It may also include additional annotated fields and data regarding sequence and patient information. The data are made accessible through tools allowing searches on some 30 different fields with automatic exclusion of sequences such as from non-human hosts or those that are epidemiologically related (either from one patient or from a cluster of linked infections). Searching for all sequences of a particular genomic region (e.g., E1 and E2) is available with the possibility of downloading the result as an alignment. The other section of the database addresses molecular immunology and contains lists of the defined HCV epitopes that are searchable [30]. However, this part of the database has not been maintained since September 2007.

HCVIVdb, compared to these databases, is more specific with regards to its aim of displaying IRES variation data specifically and in a precise manner. It is unique in providing a centralized repository for HCV IRES mutations along with the functional consequences of these mutations. It mediates transfer and display of information about mutations in the HCV IRES gathered from well-defined published sources along with added information and analysis tools. We hope that HCVIVdb may help in functional analyses of particular HCV IRES regions or nucleotides.

Some additional databases that address the hepatitis C virus are summarized in the Additional file 1.

## Conclusion

HCVIVdb is a specialized relational database that focuses on the reported variations of the HCV IRES that have been found in patients and/or purposely introduced to the viral genome and on the impact of these variations on HCV IRES activity. The database offers insight into the functional significance of the HCV IRES domains, subdomains, regions and even individual nucleotides. The design of the database permits users to access, analyze and download relevant information through the sophisticated but user-friendly graphical interface. The HCVIVdb is an efficient and helpful tool for people working in both the HCV and IRES fields and can aid in the understanding of the IRES function, development and design of new experiments and in a targeted drug design.

## Availability and requirements

HCVIVdb is freely available at http://hcvivdb.org/. Scientists are encouraged to submit their data concerning HCV IRES mutations either through the dedicated form within the HCVIVdb web site or directly to the corresponding author. New entries will be added in batches by the database curators.

## Abbreviations

HCV, hepatitis C virus; IRES, internal ribosomal entry site; MSA, multiple sequence alignment

## Additional file

Additional file 1: A list of online databases related to the hepatitis C virus. Sections are categorized based on the content of the database, tools available and the link to the relevant website and publication. (PDF 232 kb)
