# Supplementary material for: HCVIVdb: The hepatitis-C IRES variation database
Source: BMC Microbiol. 2016 Aug 15;16:187. doi: 10.1186/s12866-016-0804-6 (PMC4986321; doi:10.1186/s12866-016-0804-6)
Supplement: Additional file 1: — A list of online databases related to the hepatitis C virus. Sections are categorized based on the content of the database, tools available and the link to the relevant website and publication. (PDF 232 kb) [file 12866_2016_804_MOESM1_ESM.pdf]

Additional file 1 A list of online databases related to the hepatitis C virus. Sections are categorized based on the content of the database, tools available and the link to the relevant website and publication.

Name	Database Content	Analytical tools	Links and references
euHCVdb: The European HCV database	HCV: protein sequence, structure and function analyses. In collaboration with Japanese (Hepatitis Virus db) and US databases (Los Alamos). Selection of computer-annotated HCV sequences based on reference genome includes genome mapping of sequences, sub-typing and 3D molecular models of proteins.	Tools: divided into static and dynamic sections<br>Static: Access to genomic regions and proteins with known 3D structures are linked to PDB.<br>Dynamic: consist of a query section, dynamic set of sequences and 3D model with variety of user-defined criteria. The available data can be exported and further analyzed by bioinformatic programs in Web servers @NPS and/or PIG.<br>Links to external sources such as EMBL	https://euhcvdb.ibcp.fr/euHCVdb/jsp/index.jsp<br>http://www.ncbi.nlm.nih.gov/pubmed/17142229
HCV pro	HCV: Virus-virus and virus-cellular protein-protein interaction, functional genomics, hepatocellular carcinoma (HCC)-related gene expression and molecular data.<br>Manually verified interaction curated from the literature and databases.<br>Data on Hepatocellular carcinoma related gene expression<br>on genes encoding cellular proteins.	Tools available: protein selection (HCV and humans) chromosomal numbers and evidence that includes experiment type for concluding the interactions along with PubMed ID. Additionally, HCVpro, BIND and VirusMint IDs.	http://cbrc.kaust.edu.sa/hcvpro/index.php<br>http://www.ncbi.nlm.nih.gov/pubmed/21930248/<br>Updated

HCVIVdb: HCV IRES variation database	Focused on the variations that have been found in the HCV IRES sequence across various genotypes and subtypes. The datasets from studies have been collated for the users to understand the function of HCV IRES associated with diverse mutations described in different domains.<br>Allows direct comparative and functional analysis.	Tools that provide extensive search into the variation entries.<br>HCVIVdb entries are grouped into further categories based on genotypes, naturally occurring or engineered, location of occurrence, mutation type, range of translation efficiency and original publication.<br>The data related to any category can be retrieved and downloaded.	http://hcvivdb.org/references.php<br>http://www.ncbi.nlm.nih.gov/pubmed/25352252
HCV-LANL	HCV sequences with details of isolate and patients.<br>Association of HCV variants with infection. Individual and epidemiological outbreaks.	Retrieve data (sequences, clinical information)<br>Tools for sequence variation analysis.	http://hcv.lanl.gov/content/index<br>http://www.ncbi.nlm.nih.gov/pubmed/15377502<br>Part of database that addresses immunology is not maintained since 2007.
HepSEQ-Research Database System	Hepatitis B infection: molecular, clinical, epidemiological, nucleotide sequence and mutational features.<br>Information on HBV genotype and mutational recognition with known clinical impact.<br>Sequence homology searches.<br>All the data are collected from participating centers consisting of virological, clinical, and epidemiological locations, and nucleotide sequences are manually checked and uploaded to the database.	Tools provided: SeqMatcher, Genotyper, Gene Mutation, Mutation Annotator.<br>SeqMatcher: Sequence homology searches.<br>Genotyper: Identify HBV genotypes.<br>Gene Mutation: Identify mutations in HBV coding regions.<br>Mutation annotator: Annotate sequences known to be linked to anti-viral resistance.	http://www.hepseq.org/Public/Web_Front/main.php<br>http://www.ncbi.nlm.nih.gov/pubmed/17130143
Immune Epitope	Characterizing; human, non-human, primates.	Provides intrinsic structural and phylogenetic features.	Epitopes curated manually.<br>http://www.iedb.org/home_v3.php

Database and Analysis Resource	Antibody, T-cell epitopes involved in infectious disease, allergy, auto-immunity, HCV epitopes, experimental and self-antigens.	Epitope interaction with host immune system.<br>Tools to query database and analyze epitope information. Also assists in prediction of B-cell and T-cell epitope.	http://www.ncbi.nlm.nih.gov/pubmed/25300482
PhEVER	Interaction; virus-virus, virus-host and drive novel functions through the exchanges of their genetic material (lateral gene transfer).<br>Evolutionary (Viral and protein evolution) and phylogenetic information.<br>Homologous gene family b/w<br>(a) different virus sequences<br>(b) viral seq and seq from cellular organisms.<br>Clustering of homologous protein families.	Complete genomes and their annotations were taken from: (Ensembl, Genome Reviews and RefSeq Viral).<br>Detect sequence alignments and homologies.<br>2426 non-redundant viral genomes.<br>1007 non-redundant prokaryotic genomes.<br>43 non-redundant eukaryotic genomes.<br>Tool to search and analyze horizontal gene transfer.	http://pbil.univ-lyon1.fr/databases/phever/index.php<br>http://www.ncbi.nlm.nih.gov/pubmed/?term=PMC3013642
RNA virus Database	Genome organization.<br>Identify submitted nucleotides.<br>Translated genome sequences for all species.	Analytical tools for 938 known species of RNA virus.<br>Multiple whole-genome alignments.<br>Guidance to other web sources.	http://bioafrica.mrc.ac.za/rnavirusdb/<br>http://www.ncbi.nlm.nih.gov/pubmed/?term=18948277<br>The database currently has 1062 viruses (June 2010)<br>Page layout last updated 17 February 2011
VBRC: Viral Bioinformatics resource center	Sequence data.<br>Curation: Viral genomes and genes<br>Arenaviridae, Bunyaviradae, Filoviradae, Paramyxoviradae, Poxviridae and Togaviridae family including HCV.	Tools that provide searchable and comprehensive analysis of gene function with relation of genotype to phenotype and pathogenesis.	For updated sequence data and analytical tools, please visit the ViPR BRC: (http://www.viprbrc.org)

VIDA (The virus database)	Characterize animal virus open reading frame sequences.<br>Homologous protein families from complete and partial viral genomes.<br>Provides means to study evolution and function in viruses.<br>Further cataloging of homologous protein families into various functional classes.<br>Virus families: Arteriviridae, Coronaviridae, Herpesviridae, Papillomaviridae, Poxviridae.	Complete and partial genomes of viral families taken from GenBank and filtered.<br>Links to PDB and CATCH for protein structures.<br>Alternative ways to search for a query in database.	http://www.biochem.ucl.ac.uk/bsm/virus_database/VIDA3/VIDA.html<br>http://www.ncbi.nlm.nih.gov/pubmed/11125070<br>Appears that it is no longer being updated
VIRALZONE	Virion structure, replicative cycle, host-virus interaction.<br>Molecular biology and epidemiology for each virus genus with links to proteome UniportKB.<br>133 viral ontology pages.	Illustrations, text and PubMed references.<br>Links to many databases.<br>List of many annotated proteins in UniportKB.	http://viralzone.expasy.org/<br>http://www.ncbi.nlm.nih.gov/pubmed/23193299
